# Ambulatory seizure forecasting with a wrist-worn device using long-short term memory deep learning

**DOI:** 10.1038/s41598-021-01449-2

**Published:** 2021-11-09

**Authors:** Mona Nasseri, Tal Pal Attia, Boney Joseph, Nicholas M. Gregg, Ewan S. Nurse, Pedro F. Viana, Gregory Worrell, Matthias Dümpelmann, Mark P. Richardson, Dean R. Freestone, Benjamin H. Brinkmann

**Affiliations:** 1 Departments of Neurology and Biomedical Engineering, Mayo Foundation, Rochester, MN USA; 2 School of Engineering, University of North Florida, Jacksonville, FL USA; 3 Seer Medical Inc., Melbourne, VIC Australia; 4 Department of Medicine, St. Vincent’s Hospital Melbourne, University of Melbourne, Melbourne, VIC Australia; 5 Institute of Psychiatry, Psychology and Neuroscience, King’s College London, London, UK; 6 Faculty of Medicine, University of Lisbon, Lisboa, Portugal; 7 Department of Neurosurgery, Epilepsy Center, Medical Center – University of Freiburg, Faculty of Medicine, University of Freiburg, Freiburg, Germany; 8 Departments of Neurology and Biomedical Engineering, Mayo Foundation, Alfred 9-441C, SMH, 200 First Street SW, Rochester, MN 55905 USA

**Keywords:** Epilepsy, Learning algorithms, Neurology

## Abstract

The ability to forecast seizures minutes to hours in advance of an event has been verified using invasive EEG devices, but has not been previously demonstrated using noninvasive wearable devices over long durations in an ambulatory setting. In this study we developed a seizure forecasting system with a long short-term memory (LSTM) recurrent neural network (RNN) algorithm, using a noninvasive wrist-worn research-grade physiological sensor device, and tested the system in patients with epilepsy in the field, with concurrent invasive EEG confirmation of seizures via an implanted recording device. The system achieved forecasting performance significantly better than a random predictor for 5 of 6 patients studied, with mean AUC-ROC of 0.80 (range 0.72–0.92). These results provide the first clear evidence that direct seizure forecasts are possible using wearable devices in the ambulatory setting for many patients with epilepsy.

## Introduction

Despite optimized medication therapy, resective surgery, and neuromodulation therapy, many people with epilepsy continue to experience seizures. Half or more of patients who undergo resective surgery for epilepsy have eventual recurrence of seizures^1, 2^, and devices for neuromodulation rarely achieve long-term seizure freedom^3, 4^. People living with epilepsy consistently report the unpredictability of seizures to be the most limiting aspect of their condition^5^. Reliable seizure forecasts could potentially allow people living with recurrent seizures to modify their activities, take a fast-acting medication, or increase neuromodulation therapy to prevent or manage impending seizures. Accurate seizure forecasts have been demonstrated using invasively sampled ultralong-term EEG in ambulatory canine^6–8^ and human subjects^9–14^, including a prospective study with a dedicated device^11^. However, invasive devices may not be acceptable for some patients with epilepsy, and no clinically available invasive device currently has the capability to sample and telemeter data needed for seizure forecasting. Hence there is presently great interest in forecasting seizures using wearable or minimally invasive devices. Deep learning approaches have shown promising performance for a variety of difficult applications^15^, including seizure forecasting^7^. In particular these “end-to-end learning” methods are attractive for seizure forecasting given the challenges of identifying salient features in ultra-long term time-series data, and the heterogeneity in time series data characteristics between different patients. The power and capability of deep learning algorithms trained on very large datasets hold promise to enable applications not previously believed possible, and may open the door to seizure forecasting with noninvasive sampling devices.

Many challenges exist in designing a reliable system for forecasting seizures from noninvasively recorded data. Training, testing, and validating a forecasting algorithm requires ultra-long duration recordings with an adequate number of seizures. Additionally, concurrent video and/or EEG validation of seizures in an ambulatory setting over months to years is logistically difficult, and is not possible using conventional in-hospital monitoring methods. Self-reported seizure diaries are the most accessible validation, but the poor reliability of such diaries is widely recognized^11, 16^. Performing device studies on in-hospital patients with concurrent video-EEG validation is logistically feasible, but such studies are expensive, and limited in duration, and restrict normal daily activities which could produce false alarms, such as exercise, brushing teeth, or other activities. Because of these challenges an ILAE-IFCN working group recently published guidelines^17^ for seizure detection studies with non-invasive wearable devices, but few studies achieve phase 3–4 evidence in an ambulatory setting^18^. In studies of seizure forecasting it is imperative that ambulatory data including the full range of normal activities be included in the training, testing, and validation sets.

Seizure prediction with wearable devices was recently investigated in a cohort of in-hospital patients^19^ using a cross-patient deep learning algorithm on data recorded from Empatica E4 devices. The dataset was comprised of multiday recordings from 69 epilepsy patients (28 female, duration 2311.4 h, 452 seizures). In a leave-one-patient-out cross-validation approach, they achieved better than chance prediction in 43% of patients, with no difference in performance between generalized and focal seizure types. It has also been shown that seizure occurrence can be modeled as circadian or multiday patterns of seizure risk over long periods^20, 21^, and these patterns may be used to forecast seizures^22^. Using a mobile electronic seizure diary application^21^ seizure forecasts calculated based on circadian and multiday seizure cycles using data from 50 application users produced accurate forecasts for approximately half the cohort. Long-term cycles of seizure risk offer complementary information to direct forecasting of seizures, and signals from wearable fitness trackers have been shown to have value in identifying circadian and multidian cycles of seizure risk^23^.

This study aimed to develop a wearable seizure forecasting system for ambulatory use, and to evaluate the forecasting performance relative to seizures identified with concurrent chronic intracranial EEG (iEEG).

## Methods

This study was reviewed and approved by the Mayo Clinic Institutional Review Board (IRB 18-008357), and all study methods were performed in accordance with all applicable regulations and guidelines and the declaration of Helsinki. Informed consent was obtained from all study participants and/or a legal guardian before enrollment in the study. Patients were recruited who had drug-resistant epilepsy and were treated with a responsive neurostimulation device (NeuroPace RNS(R) system; NeuroPace Inc., Mountain View, CA) implanted as part of their clinical care. This RNS system provides chronic iEEG monitoring, and clinician-defined detectors trigger storage of iEEG timeseries epochs for suspected seizure activity, and trigger therapeutic stimulation. Patients upload RNS data as part of routine clinical care, and these iEEG clips were reviewed for seizure activity by a board-certified epileptologist. The implanted device is capable of storing a limited amount of raw iEEG data—for our subjects and setting parameters the RNS device stored up to eight iEEG clips between uploads, and any additional recorded clips would write over previous data. Hence we recruited only patients with eight or fewer stored clips at each upload, and we confirmed the timestamps of the clips covered the entire upload interval. Furthermore we required recruited subjects to have stored clips without seizure activity (i.e. false positives) on the device to ensure we were not missing seizure events. Of note, the trigger on the RNS device for stimulation and for iEEG storage are different, and the number of stimulations per day is typically far greater than epochs of raw iEEG storage^24^. Patients who had their primary epilepsy care at Mayo Clinic Rochester MN, Scottsdale AZ, or Jacksonville FL were identified and screened for participation based on their ability to operate a wearable device and the quality and coverage of their EEG data recordings. Each patient’s primary epileptologist was consulted to identify significant psychiatric, social, or other clinical factors that counter indicated involvement in the study before enrollment.

Patients were given two wrist-worn recording devices (Empatica E4, Empatica Inc., Boston MA) and a tablet computer to record and upload data daily to the Empatica cloud^25^. Patients were instructed to wear one wristband while the second band charged and synchronized data via the tablet computer, and to exchange devices at a specific time each day. Patients otherwise went about their usual daily activities. The Empatica E4 device records physiological data including 3-axis accelerometry (ACC), blood volume pulse (BVP) measured by photoplethysmography (PPG), electrodermal activity (EDA), and temperature (TEMP). A minimum of approximately 6 months of recorded wearable and concurrent iEEG data was required for inclusion in the study^25^.

**Figure 1:**
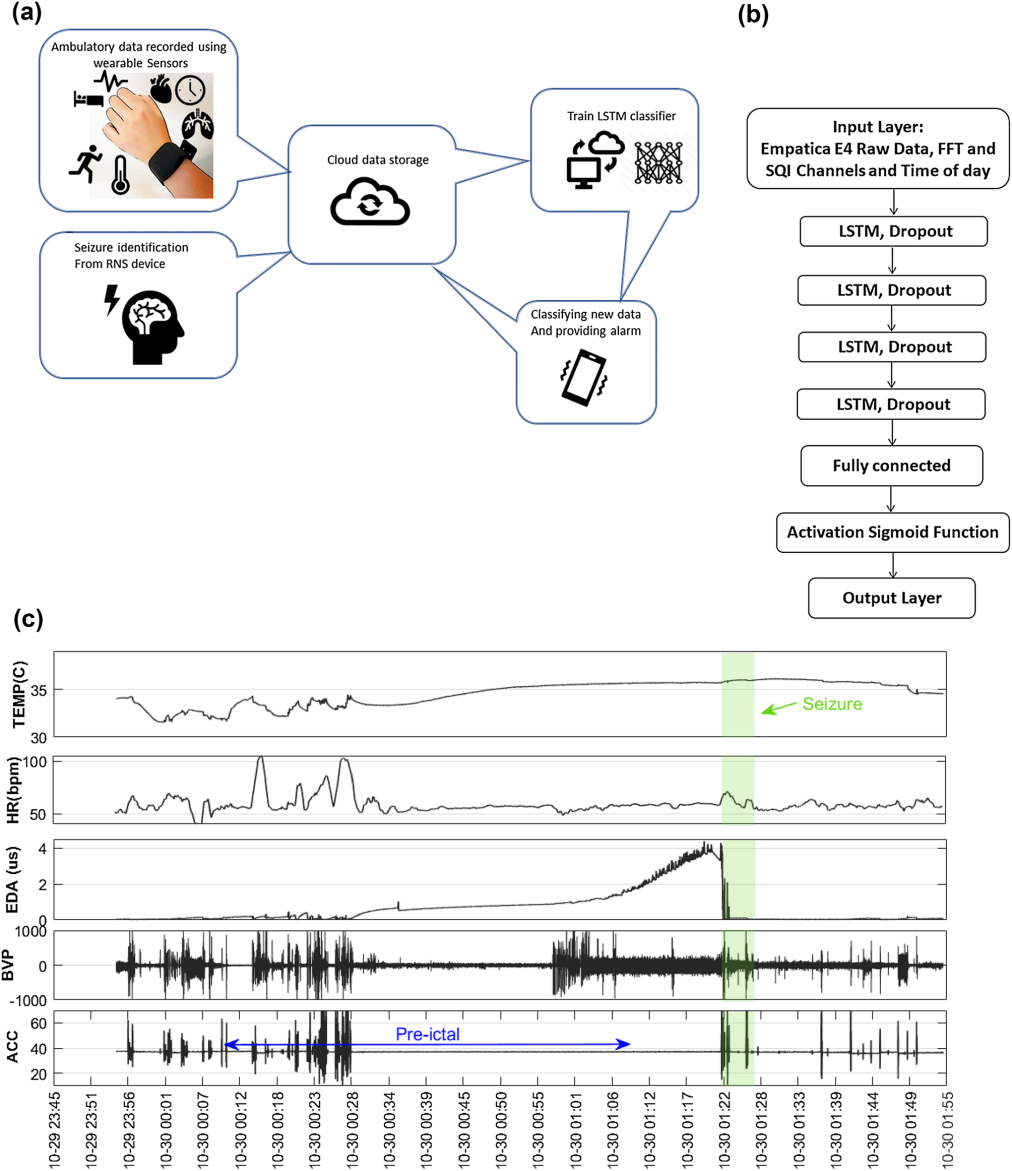
(**a**) Data flow diagram of ambulatory data recorded by wearable sensors, transferred via cloud storage, and analyzed using deep learning. Ambulatory data recorded using Empatica E4 wristbands was uploaded regularly to cloud storage by patients and was downloaded by study staff. Patients uploaded RNS data as part of routine clinical care, and the iEEG clips were reviewed for seizure activity. (**b**) Architecture of machine learning classifier with 4 LSTM layers, 128 hidden nodes, one dropout layer after each LSTM layer with a dropout rate of 0.2, a fully connected layer, and an output layer to generate the classification output using a sigmoid activation function. (**c**) Raw wearable data plotted showing accelerometry, EDA, temperature, and blood volume pulse with derived heart rate for a preictal segment from 75 to 15 min before the approximate seizure time (green).

A long short-term memory (LSTM) Recurrent Neural Network (RNN) algorithm was designed with 4 LSTM layers, 128 hidden nodes, one dropout layer after each LSTM layer with a dropout rate of 0.2, a fully connected layer, and an output layer to generate the classification output using a sigmoid activation function (Fig. 1b). The algorithm was trained on 60-s data segments selected from each recording. To ensure the algorithm was performing seizure forecasting rather than early seizure detection, and to account for potential misalignment between the clocks in the wearable and implanted devices and the potential inexact timing of the seizure onset recorded by the device^26, 27^, one-hour preictal data epochs were defined with a set-back of 15 min before the seizure onset recorded by the implanted EEG device. Lead seizures were defined as seizures separated from preceding seizures by at least 4 h, and clustered (i.e., non-lead) seizures were excluded from analysis to avoid artificially inflating results. A typical architecture of the whole process including collecting wearable data, annotations, designing the deep learning classifiers, providing alarm, classifier architecture and also a sample of recorded wearable data are shown in Fig. 1.

### Signal quality evaluations for ACC, BVP and EDA signals

To have a measure of the quality of data, signal quality metrics were calculated according to techniques reported previously^27^. For EDA, the rate of amplitude change in concurrent one-second windows was calculated. Sharp changes in signal amplitude of more than a 20% increase or 10% decrease per second, were considered artifact due to subject motion or poor electrical contact with the skin. The signal quality of BVP was assessed by calculating the spectral entropy for 1-min data segments averaged over non-overlapping 4-s windows. Epochs with entropy below 0.9 were considered good quality. The signal quality metric for root-mean-square of ACC data was measured as the ratio of narrowband physiological (between 0.8 Hz and 5 Hz) and broadband (0.8 Hz to Nyquist frequency) spectral power. The power of the periodogram was calculated for non-overlapping 4 s segments, and average values over consecutive 1-min segments were calculated.

### Training and testing data

Accelerometry (ACC), blood volume pulse (BVP), electrodermal activity (EDA), temperature (TEMP) and heart rate (HR) signals, recorded with Empatica E4 device with sampling frequencies of 32, 64, 4, 4, 1 Hz respectively, were up-sampled to 128 Hz to facilitate analysis. Signal quality metrics were computed^27^ for ACC, BVP and EDA and were provided to the LSTM algorithm to allow the algorithm to account for data quality in its classifications. The time of day (encoded as the hour portion of the 24-h time) and Fourier transforms of BVP, EDA, TEMP, HR, and root mean squared (RMS) accelerometry, calculated and used as inputs to the LSTM. The physiological time-series signals (ACCX, ACCY, ACCZ, ACCMag, BVP, EDA, TEMP, HR), their Fourier transforms (FFT(ACCMag), FFT(BVP), FFT(EDA), FFT(TEMP), FFT(HR)), the SQI values for ACCMag, BVP, and EDA and time of day were formed 17 channels (Fig. 1b). To compensate for the unbalanced interictal/preictal data ratio (range 1 to 6) in training, noise-added copies of the preictal data segments were generated and used to equalize the training data classes. Additive random noise was generated from a uniform distribution over [0, 1), multiplied by the median of the segment.

All training data were taken from the early part of each patient’s recording, while testing results were computed on the later portions of the patient’s data. The cutoff point between training and testing data was chosen in each patient’s recording at approximately 1/3 of the total record duration, and was adjusted to ensure including preictal segments from at least four seizures for training to provide 240 60-s preictal segments (Some patients continued acquiring data during the analysis phase of the study, and newly acquired data was incorporated into the final testing analysis for these patients). Consecutive non-overlapping 60-s data epochs were extracted and preprocessed before being used in the LSTM algorithm. The training dataset was normalized by subtracting its mean and dividing by the standard deviation (z-scoring). The training data mean and standard deviation were similarly used to normalize the test dataset. This setup approximates a seizure forecasting system that could be applied prospectively, as future information was strictly excluded from the algorithm testing phase. The area under the Receiver Operating Characteristic (AUC) was used to evaluate the classifier performance on test data segmented into 1-h data epochs. Mean classifier probability values were calculated for groups of five consecutive 1-min segment, and the maximum probability across each 60-min interval was calculated.

In order to assess the relative contributions of each signal from the wearable device (ACC, BVP, EDA, Temperature, HR and Time of the day) to the overall seizure forecast, the classifier was retrained and retested with each input signal and related channels removed in turns, and the resulting AUC subtracted from the full algorithm’s AUC. For example, to measure the importance of the accelerometer signal, ACCX, ACCY, ACCZ, ACCMag, the Fourier transform of ACC and it’s SQI were omitted and the LSTM was retrained and performance measured. Due to the random initial assignment of weights in the classifier, the algorithm was trained and tested five times for each signal, and the average AUC difference was calculated.

### Statistical evaluation

To measure whether the prediction algorithm performs significantly better than chance, the statistical test described in^28^ was used. The authors assumed the probability of a preictal alert follows a Poisson probability distribution, λ_w_Δt in a short interval of duration Δt, where λ_w_ is the Poisson rate parameter. Assuming the successful prediction of n out of N seizures, the two-sided p-value is the probability of observing a difference between the classifier and a Poisson random predictor. As an additional validation of our testing approach and statistical assessment of our results, we randomized the seizure times for each subject and recalculated the AUC for the LSTM output. This was repeated 100 times for each subject, and the mean and standard deviation of the AUCs were recorded. Random seizure times were generated^29^ such that the total number of seizures, and the distribution of intervals between consecutive seizures were constant. We calculated the sensitivity of the random predictor with an equal time in warning, and reported its sensitivity difference with the classifier result^30^.

## Results

Six patients were successfully recorded for approximately six or more months (median 220 days) with good quality EEG and wearable data. One patient’s wearable device developed a defective BVP sensor during the study and was replaced after approximately 6 weeks. The faulty BVP data epochs (interleaved days) were excluded from training, testing, and further calculations. The demographics, epilepsy characteristics, sensor placement, and available data for the six patients studied are described in Table 1.

**Table 1:** Cohort demographics, epilepsy characteristics, and data characteristics.

Age	Gender	Age of onset	Wristband location	Epilepsy type	Epilepsy localization	Predominant seizure semiology	Anti-seizures meds (mg/day)	Median stims per day	Participation (days)	Recorded data (days)	Training data days (seizures)	Test data days (seizures)
21	F	15	Left wrist	Focal onset impaired awareness seizures, and focal to bilateral tonic–clonic seizures	Left temporal	Initial sense of fear, followed by receptive aphasia, subjective sensation of feeling warm and diaphoretic, and gagging and retching. May progress to generalized convulsions	Levetiracetam XR, 4500	1640	242	207	55 (4)	152 (3)
42	F	9	Left wrist	Focal onset impaired awareness seizures	Left frontocentral	Eyes moved to the left side with brief twitching, followed by flexion of upper extremities	Felbamate 2700, Lamotrigine 600, Levetiracetam 4000	340	200	188.3	85 (7)	103 (9)
38	F	20	Right wrist	Focal onset impaired awareness seizures, and focal to bilateral tonic–clonic seizures	Left parietocentral and right frontocentral (L > R)	Vision difficulties, swallowing difficulties, and speaking difficulties prior to seizure onset followed by right extremity jerking and behavioral arrest with loss of awareness. May progress to generalized convulsions	Gabapentin 1200, Levetiracetam 2500, Vimpat 600	898	236	193.3	96 (17)	97 (11)
41	M	4	Left wrist	Focal onset impaired awareness seizures, and focal to bilateral tonic–clonic seizures	Left temporal	Unresponsive with lip smacking, impaired awareness, hand movements and may progress to generalized convulsions	Qudexy XR 100, Levetiracetam 6000	484	370	327.5	140 (4)	187.4 (6)
53	M	18	Right wrist	Focal onset aware seizures, and tonic–clonic seizures	Independent bitemporal	Staring with speech arrest and no movements, possible posturing of extremity, and automatisms. Rare convulsive seizures	Lamotrigine 200	1566	265	252.2	66 (31)	185.3 (171)
27	M	12	Left wrist	Focal onset impaired awareness seizures	Left temporal	Staring and unresponsiveness, sometimes with laughter-like vocalization	Lacosamide 200	1542	166	152.5**	76 (7)	76.2 (8)

The median (range) age was 39.5 (21–53) years, and the cohort consisted of three males and three females. Lead seizures are reported and clustered (< 4 h separation) seizures were not included.

**662 h of recording excluded due to bad BVP and HR signals.

### Data quality

The proportion of EDA and BVP data with good quality according to our calculated SQI metrics are reported in Table 2.

**Table 2:** Intra-subject performance of forecasting algorithm.

Age	Gender	Good quality BVP (%)	Good quality EDA (%)	AUC-ROC	Sensitivity	Time in warning (H/day)	P-value	Mean pre-seizure alert (minutes)	Random AUC mean (st. dev.)	Improvement over chance
21	F	77	54	0.88	0.66	3.4	0.049	30	0.54 (0.25)	0.45 (0.32)
42	F	91	77	0.75	0.66	7.04	0.010	42	0.50 (0.11)	0.37 (0.16)
38	F	78	63	0.75	0.72	7.2	0.002	29	0.50(0.08)	0.40 (0.13)
41	M	79	69	0.92	0.66	0.9	0.0002	28	0.48 (0.22)	0.63 (0.08)
53	M	76	85	0.50	–	–	–	–	0.47 (0.023)	
27	M	82	88	0.72	0.62	6.4	0.024	36	0.51 (0.12)	0.33 (0.18)

P-values were computed using the method described by Snyder et al.^28^. The signal quality metrics were calculated according to techniques reported previously^27^ and the percentage of the data with good quality was reported for EDA and BVP signals. Random AUC values were calculated by randomizing the seizure times for each subject and repeating the scoring 100 times for each subject. The sensitivity difference between results and random output was calculated at the same Time in Warning.

### Forecasting

**Figure 2:**
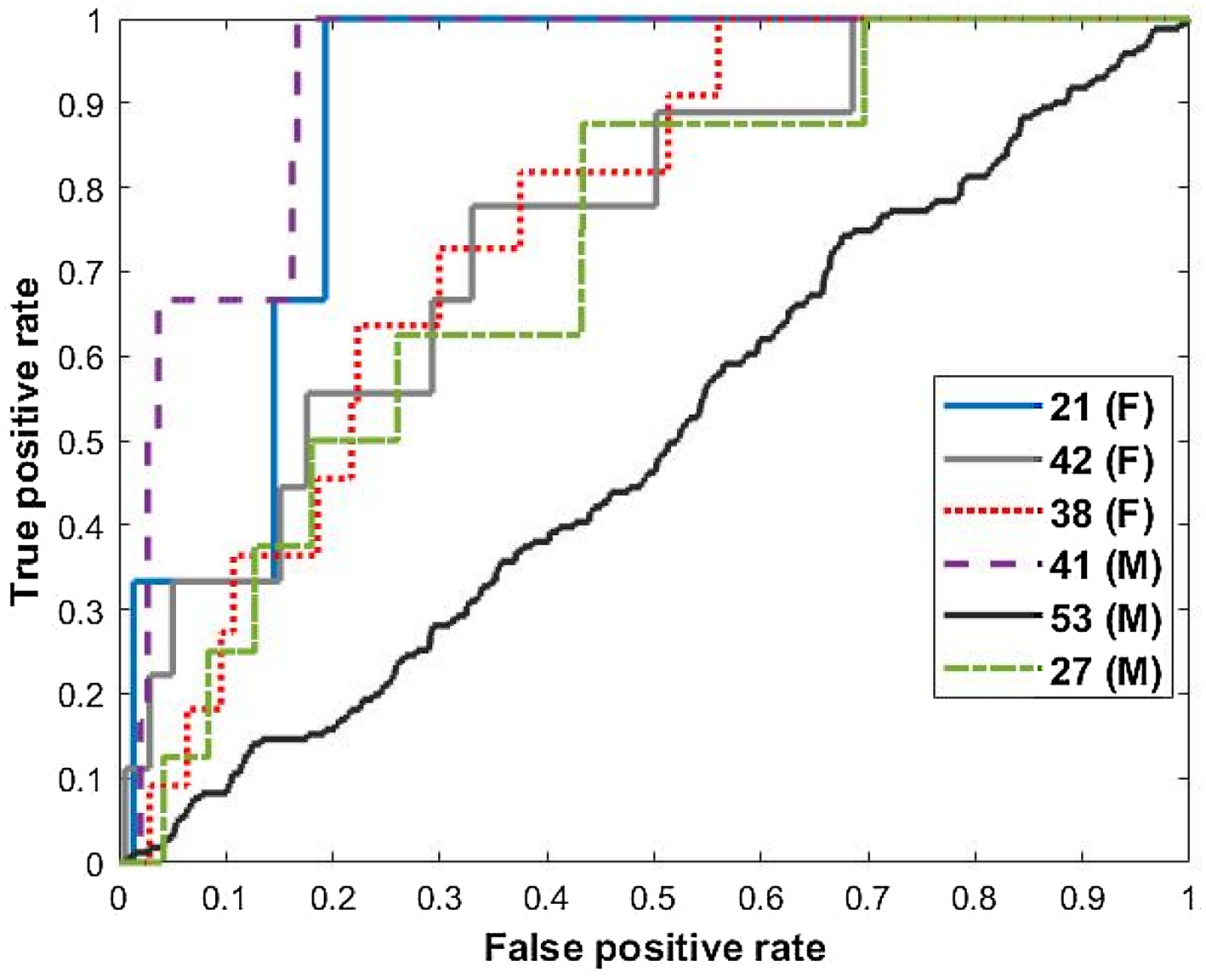
The receiver operating characteristic (ROC) curve for ambulatory patients (Table 2). Five of six patients analyzed achieved seizure forecasts significantly more accurate than a chance predictor, with mean AUC-ROC of 0.80 (range 0.72–0.92).

Five of the six patients analyzed had seizure forecasts significantly more accurate than a random predictor, according to the criteria described by Snyder et al.^28^. A mean (st. dev.) AUC of 0.75 (0.15) was achieved across the cohort using the LSTM classifier, compared to 0.48 (0.01) in aggregate using randomized seizure times. Seizure alerts occurred on average 33 min before the EEG-recorded seizure onset across the cohort. Full results are reported in Table 2. The ROC curve is shown in Fig. 2.

### Feature importance

**Figure 3:**
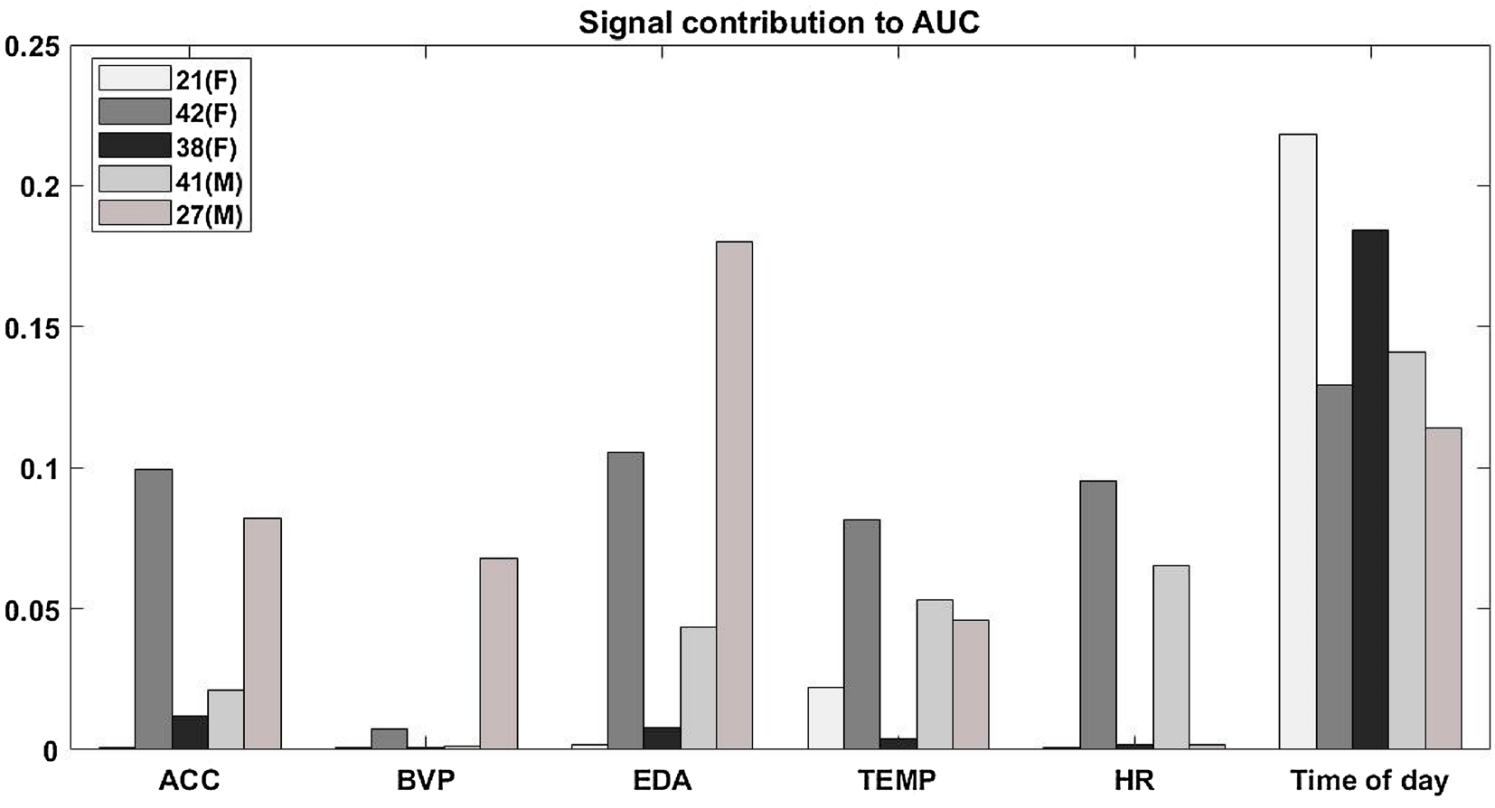
Influence of each signal and its related features on classifier performance. The algorithm was trained and tested five times with each signal removed, and the average AUC difference from the full result was calculated.

The relative contribution of individual signals is shown in Fig. 3. Time of the day was the most consistently important across all patients, but most patients’ results also showed high reliance on ACC, EDA, and TEMP.

## Discussion

This study presents Phase 2 retrospective evidence according to the IFCN guidelines^17^ of successful forecasting of seizures using a wearable device in a cohort of ambulatory patients. Ultra-long-term (multiple months) ambulatory studies for seizure detection and forecasting are critical to ensure training and testing occur over a large dataset that captures the full range of normal activities. Such ambulatory studies are challenging given the need for concurrent EEG validation over long periods of time in a home environment, the associated technical difficulties of timestamp synchronization and remote support^25^, and the burden on patients^31, 32^. These challenges drove our choice to use a conservative pre-seizure set-back of 15 min, in contrast to prior studies with a single dedicated intracranial EEG device which have used a 5-min set-back to eliminate any ambiguity with annotation of the actual onset of the seizure^9–11^. During an earlier in-hospital phase of data acquisition with the Empatica wearable device, we found timestamp errors of up to 2 min per 24-h period^25, 27^, and given the limited spatial coverage and data record length of the implanted EEG device, additional allowance was warranted. The 15-min setback represents a worst-case scenario and provides a conservative estimate of our system’s performance.

A further limitation of ambulatory data collection in this study was the infeasibility of recording seizure semiology to correlate with EEG, either through video or other measures due to privacy concerns and the inability to fully cover the patient’s environment. It is possible that accounting for seizure semiology in training data could improve accuracy, and in particular whether a recorded seizure had clinical manifestations. Data quality is also a challenge in ambulatory, in-home studies, and further improvements in sensor design are needed to minimize artifacts due to motion and poor device fit. Device comfort is also an important factor, as a comfortable device is more likely to be worn snugly on the wrist, thereby minimizing movement artifacts. Due to the ultra-long term recording durations in this study, device laterality was chosen to maximize comfort, and it is possible that placing devices on the wrist contralateral to seizure onset might improve results. Battery life and charging are also considerations for long-term acceptability and adherence, as a prospective seizure forecasting system is not likely to be compatible with our approach of exchanging devices daily.

The RNS device has multiple on-board detectors, and stimulation is typically delivered with hypersensitive detector settings resulting in hundreds to thousands of stimulations per day^24^, consistent with our data in Table 1. These detections are primarily interictal discharges, and separate detectors for long episodes or signal saturation are used to store EEG clips and identify seizures. Without full 24/7 EEG^33^ we can’t be entirely sure no seizures were missed, but storage of false positive clips on the device, and agreement with the patient’s reported seizure burden suggests a high degree of accuracy.

Previous studies of forecasting with invasive EEG have shown poorer performance with an increased number of seizures^11^, and this pattern was also apparent in our data. The one patient for whom forecasting did not perform significantly better than a random predictor had multiple seizures each day, while the other subjects had seizures less frequently. It is possible that the choice of a four-hour interval to define lead seizures may not be adequate to allow the patient to fully return to a baseline state after a previous seizure, and this may confuse the classifier’s characterization of the baseline interictal state. The time of day input feature showed the highest contribution to the results of most patients studied, suggesting that patients with a strong circadian pattern may have better results than those without. Other measured signals contributed substantially as well to the overall accuracy, but the relative contribution varied by patient.

The accuracy of forecasts required to be clinically useful varies based on the intended use of the forecast. For neuromodulation, where little or no penalty for temporarily increased stimulation due to false alarms exists, multiple alarms daily may be acceptable. For administration of fast-acting medications, false alarms may be acceptable if the overall medication dose is better targeted toward periods of high seizure risk. False alerts are less acceptable in caregiver alert use cases, where caregiver fatigue and annoyance may lead to discontinued use of the device^34^. The level of performance reported in the present study may be acceptable for some applications, but continued improvement in accuracy, and confirmation in prospective, real-time studies are needed to advance this application. We did not attempt progressive retraining of our algorithm in this study, as has been used in some iEEG forecasting applications^7^, and this approach could improve accuracy. Reliable seizure detections with the device or reliable seizure reports would be necessary to facilitate this in a real-world application, and both of these approaches have associated challenges presently.

## Conclusions

This preliminary study in a small cohort has demonstrated seizure forecasting using a noninvasive wrist-worn multimodal sensor significantly better than a random predictor in ambulatory ultra-long-term recordings of patients with epilepsy for the majority of patients studied. Wearable data was recorded in an ambulatory setting during normal activity with concurrent EEG validation of seizure events. Five of six patients analyzed achieved seizure forecasts significantly more accurate than a chance predictor, and seizure alerts in these five patients provided ample warning time to administer fast-acting medication or to increase neuromodulation therapy. This is the first study reporting successful seizure forecasting with noninvasive devices in ultra-long-term recordings in freely-behaving humans outside the clinical environment.
